# Efficacy of adjunctive topical liposomal clarithromycin on systemic Glucantime in Old World cutaneous leishmaniasis: a pilot clinical study

**DOI:** 10.3389/fphar.2023.1280240

**Published:** 2023-11-07

**Authors:** Atousa Hakamifard, Reza Radmehr, Fatemeh Sokhanvari, Fatemeh Sherkat, Amirali Hariri, Jaleh Varshosaz, Zabihollah Shahmoradi, Awat Feizi, Bahareh Abtahi-Naeini, Mahsa Pourmahdi-Boroujeni

**Affiliations:** ^1^ Department of Infectious Diseases, Cancer Prevention Research Center, School of Medicine, Isfahan University of Medical Sciences, Isfahan, Iran; ^2^ Department of Infectious Diseases, Hormozgan University of Medical Sciences, Bandar Abbas, Iran; ^3^ Skin Diseases and Leishmaniasis Research Center, Isfahan University of Medical Sciences, Isfahan, Iran; ^4^ Faculty of Pharmacy, Debrecen University, Debrecen, Hungary; ^5^ Pharmaceutical Biotechnology, School of Pharmacy and Pharmaceutical Sciences, Isfahan University of Medical Sciences, Isfahan, Iran; ^6^ Drug Delivery System Research Center, Department of Pharmaceutics, School of Pharmacy, Isfahan University of Medical Sciences, Isfahan, Iran; ^7^ Department of Dermatology, School of Medicine, Isfahan University of Medical Sciences, Isfahan, Iran; ^8^ Department of Biostatistics and Epidemiology, School of Health, Isfahan University of Medical Sciences, Isfahan, Iran; ^9^ Pediatric Dermatology Division of Department of Pediatrics, Imam Hossein Children’s Hospital, Isfahan University of Medical Sciences, Isfahan, Iran; ^10^ Student Research Committee, Isfahan University of Medical Sciences, Isfahan, Iran

**Keywords:** leishmaniasis, cutaneous leishmaniasis, Glucantime, liposomal clarithromycin, clarithromycin

## Abstract

**Aim:** This study aimed to investigate the effects of topical liposomal clarithromycin in combination with *meglumine antimoniate* (Glucantime^®^) on cutaneous leishmaniasis (CL) lesions.

**Methods:** This pilot, randomized, double-blinded clinical trial was conducted on patients with CL lesions. Patients were randomly assigned to two groups: the first group received liposomal clarithromycin in combination with Glucantime for 28 days, while the second group received Glucantime and a placebo. Afterward, patients were followed up at 1.5, 3, and 6 months after treatment initiation and were evaluated for recovery time, induration, and size of the lesions.

**Results:** Sixty patients with CL lesions were divided into two separate groups with 30 members each and were examined. Within-group analysis revealed that recovery time in the clarithromycin group was 26.65 ± 5.12 days, while in the placebo group, it was 32.84 ± 24.43, which was statistically insignificant (*p* = 0.18). Lesion size comparison in the first and last follow-ups reduced in both groups: 7.73 ± 4.31 to 0.48 ± 0.50 in the clarithromycin group (*p* = 0.006) and 5.47 ± 5.83 to 0.76 ± 0.88 in the placebo group (*p* = 0.03). Moreover, the size of lesions in the intervention group was significantly reduced compared to that in the placebo group (*p* = 0.02). Recognizable induration reduction was observed in the clarithromycin group (2.60 ± 0.77 to 1.0 ± 0.00). No adverse effects attributable to clarithromycin were reported.

**Conclusion:** The administration of liposomal clarithromycin in combination with systemic Glucantime had a significant beneficial effect on reducing lesion size in leishmaniasis. Further studies on larger populations are recommended.

**Systematic Review Registration**: https://www.irct.ir/trial/46611.

## 1 Introduction

Cutaneous leishmaniasis (CL) has been re-emerging for the past two decades. Despite its incidence increasing worldwide, the lack of interest from financial donors, public health authorities, and professionals is largely due to the fact that it is rarely fatal and mainly affects people with low socioeconomic conditions. Accordingly, CL has become one of the so-called neglected diseases. Most of these lesions heal automatically with scar formation after a few months (Tiuman et al., 2011; Alvar et al., 2012; Hailu et al., 2016). However, treatment is necessary for several reasons, including weight loss, cosmetic importance, and the risk of presenting as a severe form, such as lupoid CL (Sundar and Chakravarty, 2013; Ponte-Sucre et al., 2017).

Pentavalent antimoniate compounds have traditionally been used to treat leishmaniasis and are the primary treatment for CL. Other available medications include paromomycin, miltefosine, amphotericin B, and allopurinol. However, prescription of these drugs is widely challenging due to their severe side effects, poor tolerability, narrow therapeutic window and risk of toxicity, long-term therapy, lack of efficacy, increasing rate of resistance, and high expense of specific formulation (Tiuman et al., 2011; Sazgarnia et al., 2012; Scott and Novais, 2016; Ponte-Sucre et al., 2017; Wolf Nassif et al., 2017).

Azithromycin and clarithromycin are macrolides and are suggested to have efficacy against intracellular microbes, including *Mycobacterium avium complex*, *Legionella*, *Toxoplasma gondii*, *Cryptosporidium parvum*, *Pneumocystis carinii*, *Plasmodium falciparum*, and *Leishmania* promastigotes (Balcioğlu et al., 2012; Sazgarnia et al., 2012). Topical liposomal clarithromycin has been indicated to have beneficial therapeutic effects on sensitive microorganisms and could be a suitable alternative to traditional medications for patients with mild lesions (Sazgarnia et al., 2012; Alhajlan et al., 2013).

However, the effects of liposomal clarithromycin on CL lesions have not been evaluated. Therefore, in this study, we aim to investigate the effects and complications of liposomal clarithromycin in combination with meglumine antimoniate (Glucantime^®^).

## 2 Methods and materials

### 2.1 Trial design and participants

The current study was a single-center, pilot, randomized, double-blinded clinical trial performed in 2020, from April to October, on patients with CL lesions in educational dermatology or infectious diseases clinics or leishmaniasis centers in Isfahan, Iran. The trial was approved by the Ethics Committee of the Isfahan University of Medical Science (Grant No. IR.MUI.MED.REC.1398.396). The study was conducted in accordance with the Declaration of Helsinki and subsequent revisions and was registered at the Iranian clinical trials (www.irct.ir; IRCTID: IRCT20171230038142N17). Written informed consent was obtained from all subjects before the initiation of the study.

The inclusion criteria were as follows: 1) patients aged 2–65 years, 2) diagnosed as CL cases by an expert dermatologist, 3) positive parasitology for leishmaniasis, including a positive smear of Leishman body PCR or skin biopsy, 4) lesion diameter up to 5 cm, 5) lesion count up to 5, and 6) no joint or mucosal membrane involvement. Patients were excluded if they 1) had a previous history of leishmaniasis, 2) were currently pregnant or lactating, 3) had a history of cardiac, renal, or liver problems, 4) were taking any medication that interferes with clarithromycin, 5) had any contraindication for the use of clarithromycin such as hypersensitivity to macrolide or ketolide antibiotics, and 6) declined to participate in the study. During the follow-up phase, subjects were excluded if they did not show up for follow-up visits, did not take the medication according to the study protocol, or experienced non-tolerable side effects. Choosing the criteria was based on similar studies evaluating topical liposomal medication’s efficacy on CL (Abtahi-Naeini et al., 2021; Khamesipour et al., 2022).

### 2.2 Study protocol and outcome assessment

Patients were randomized into two groups using Random Allocation software for parallel-group randomized trials introduced by Saghaei (2004). The first group received liposomal clarithromycin in combination with Glucantime, and the second group was administered Glucantime and a placebo.

All patients were followed up at three different time points during this study: 6 weeks, 3 months, and 6 months after treatment initiation. The main outcome measure was the difference in lesion size (the extent of re-epithelialization in ulcerative lesions) and lesion induration from the baseline. During the follow-up sessions, patients were examined, and re-epithelialization and lesion size were measured using a photography technique. Photographs of pre- and post-treatment were evaluated by two dermatologists who were blinded to the types of treatments. The details about any side effects or complications associated with the drug were also inquired about and collected.

### 2.3 Medications

The Glucantime treatment was based on the national standard treatment (Khamesipour et al., 2022), consisting of intralesional administration of ampules ranging from 0.2 to 2 ccs, depending on the size of the lesion, and was delivered subcutaneously and inside the lesion. This medication was administered weekly for 8 weeks to all patients. Furthermore, the first arm was instructed to apply 2 cc of liposomal clarithromycin lotion (1% clarithromycin) nightly for 28 consecutive days. The second group received equivalent volumes of normal saline as a placebo.

For this purpose, liposomal formulations of topical clarithromycin were prepared using the dehydration–rehydration technique. It was prepared in the laboratory of the School of Pharmacy at Isfahan University of Medical Sciences using the following method: 114 mg of *dipalmitoyl phosphatidylcholine* (DPPC) and 10 mg of cholesterol taken at a ratio of 6:1 M were used. They were added to a round-bottomed balloon and dissolved in a sufficient amount of chloroform–methanol (2:1). The solution dried in the rotary evaporator and turned into a thin film. Then, 1 mg of clarithromycin was dissolved in 1 mL of phosphate buffer at pH 7.4, and the aqueous solution was used to hydrate the lipid film. The resulting suspension was vortexed for 5 min and then exposed to ultrasonic waves at a frequency of 45 Hz for 2 min (in cycles of 45 s on and 10 s off). The resulting suspension was frozen and stored in the refrigerator until subsequent use. To rehydrate the suspension, 100 μL of phosphate was added, and the mixture was vortexed for 5 min at 40°C. This step is repeated three times, and finally, we added 700 μL of buffer until the final volume reached 1 mL. The free drug was separated using an ultracentrifuge. The trapped drug in liposomes was measured spectrophotometrically at 208 nm, or microbiologically, and by *Bacillus subtilis* by agar diffusion. If the measurement was to be performed using the microbial method, the bacterium was cultured overnight in cation-adjusted *Mueller–Hinton broth* (CAMHB), and a solution of 0.5 *McFarland bacterium* (1.5 × l08 CFU/mL) was prepared. The bacterial cells were then added to the autoclaved molten agar solution at 41°C, and the contents were immediately transferred to a sterile glass Petri dish (440 × 340 mm) to cover a thin layer of agar and the bottom bacterium. The liposomal clarithromycin sample was centrifuged at 12,000 g for 20 min at 4°C in the presence of Triton X-100 at a concentration of 0.2% v/v in PBS to form a pellet from which the liposomal drug was released. At this concentration, Triton does not affect bacterial growth. Walls were made with a diameter of 5 mm and filled with 25 μL of a sample of standard clarithromycin or its liposomal solutions. The *Petri* dishes were then incubated at 37° for 18 h. Then, the diameter of the growth inhibition halo was measured and repeated three times. An average of three replications was used to quantify the efficiency of clarithromycin encapsulation in liposomes. The sensitivity of the aforementioned microbial value was 0.002 mg/L, and the minimum measurable value was 0.002 mg/L, with a correlation coefficient higher than 0.99. The standard drug curve was linear in the 0.002–0.0125 mg/L range.

### 2.4 Statistical analysis

Data were analyzed using SPSS software version 24 (IBM Corp., Released 2016, IBM SPSS Statistics for Windows, Version 24.0., Armonk, NY: IBM Corp.). Continuous and categorical variables were reported as mean and frequency (percentage), respectively. Basic demographic and clinical characteristics of study participants were compared between groups using an independent sample *t*-test and a chi-squared test for continuous and categorical variables, respectively. Repeated measures analysis of variance was used as the main statistical method for comparing primary outcomes over time between the two studied groups. Through repeated measures ANOVA, changes over time for primary outcomes in each group were evaluated separately (time effect), mean changes over time between groups were compared (intervention effect), and the difference in changing over time between the two groups (interaction of time and intervention) was also evaluated. *p* < 0.05 was considered statistically significant.

## 3 Results

**FIGURE 1:**
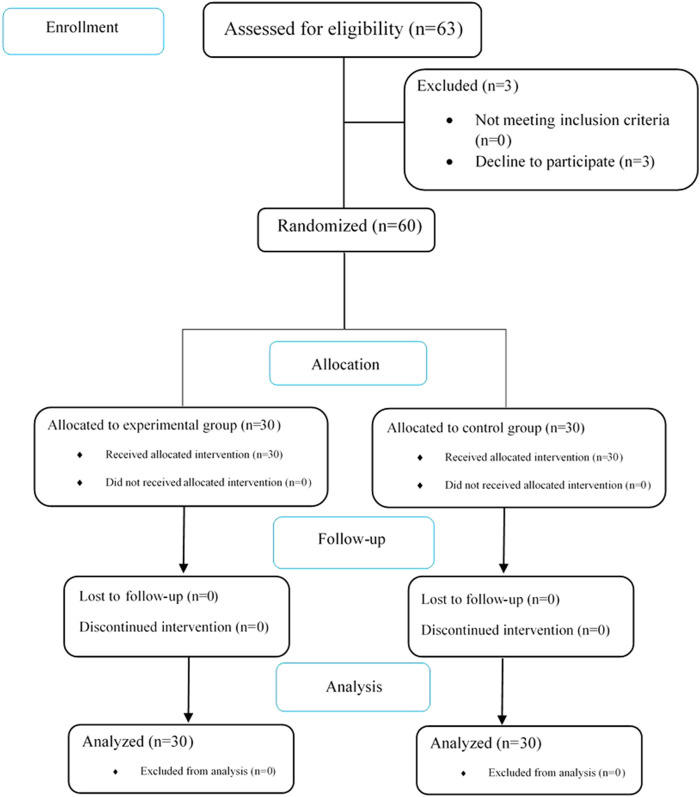
CONSORT flowchart of the study.

The present study was performed on 60 patients with CL, who met the inclusion criteria, aged 2–62 years with a mean of 27.75 years (17.05) at the Isfahan Leishmania Center. The patients were split into two groups, as shown in Figure 1. Analysis of demographic data showed that the age of the patients treated with clarithromycin was 20.60 years (12.29), and compared to the parallel group with a mean of 34.90 years (18.29), it was significantly lower (*p* < 0.001). Due to its low sensitivity and specificity, skin biopsy should be used only in atypical cases and based on the clinical course of the disease.

The location of the lesions was categorized into four groups: head and neck, trunk, upper limbs, and lower limbs. Further analysis of the frequency of distribution of the lesion site and other baseline variables, including gender and the number of lesions, was statistically the same within the groups (Table 1).

**TABLE 1:** Characteristics of the patients and investigation of the lesions in the two study groups.

Variable		Clarithromycin group (*n* = 30)	Placebo group (*n* = 30)	*p*-value
Gender	Female	3 (10%)	7 (23.33%)	0.299
	Male	27 (90%)	23 (76.67%)	
Age		20.60 (12.29)	34.90 (18.29)	0.001
Location of lesions	Head and neck	5 (16.7%)	2 (6.7%)	0.424
	Trunk	5 (16.7%)	0 (0.0%)	0.052
	Upper limb	25 (83.3%)	22 (73.3%)	0.532
	Lower limb	5 (16.7%)	12 (40.0%)	0.084
Number of lesions	1	8 (26.7%)	9 (30.0%)	0.095
	2	22 (73.3%)	17 (56.7%)	
	3	0 (0.0%)	4 (13.3%)	

Values are reported as mean (SD) and frequency (percentage) for continuous and categorical variables, respectively, resulted from independent samples. The *t*-test and chi-squared test were performed for continuous and categorical variables.

To account for the influential age difference within the groups, adjustments were made for the variable. P1, P2, and P3 resulted from repeated measures analysis of variances, and P4 resulted from an independent sample *t*-test. The mean lesion size decreased over time in both groups, but the decrease was significantly greater in the clarithromycin group than the placebo group (*p* = 0.02). Eventually, the lesion size was significantly reduced in the clarithromycin group (*p* = 0.043) (Table 2).

**TABLE 2:** Comparison of lesion size and induration.

Variable	Group	First visit	Second visit	Third visit	P1 (time)	P2 (time*intervention)	P3 (intervention)
Lesion size	1 Clarithromycin	7.73 (4.31)	3.29 (2.52)	0.48 (0.50)	0.006	0.02	0.043
	2 Placebo	5.47 (5.83)	2.35 (2.37)	0.76 (0.88)	0.03		
	P4	0.04	0.12	0.72			
Induration intensity	1 Clarithromycin	2.60 (0.77)	1.67 (0.47)	1.0 (0.00)	0.03	0.007	0.38
	2 Placebo	2.63 (0.49)	1.73 (0.94)	-	0.43		
	P4	0.87	0.80	-			

Values are reported as mean (SD). Lesion size is recorded in centimeters, and induration is recorded as intensity grading, i.e., +1, +2, and +3. According to the repeated measures analysis of variances, P1 shows the result of each approach outcome over time. According to the repeated measures analysis of variances, P2 and P3 show the comparison of within-group outcomes, and P2 considers the variable time. P4 from the independent samples *t*-test shows the comparison of within groups at each point evaluation.

Moreover, mean measurements of induration decreased over time in both groups and were statistically and clinically significant in the clarithromycin group, although there was no considerable difference between the two groups. The *p*-value for intervention was 0.38, indicating a lack of significant difference between the two groups; however, the *p*-value for intervention and time was significant at 0.007, indicating that induration decreases faster in the clarithromycin group (Table 2).

Recovery time in the clarithromycin group was 26.65 days (5.12), while in the placebo group, it was 32.84 days (24.43), which was statistically insignificant (*p* = 0.18). Additionally, patients in both groups did not report itching, dryness, swelling, redness, burning, or any other sign of allergy after treatment initiation.

## 4 Discussion

In the present study, we investigated the therapeutic effects of liposomal clarithromycin in combination with Glucantime on CL lesions. Although there were no significant differences between the two groups regarding the recovery time, the lesion size in the clarithromycin group was significantly reduced and was significantly smaller than that in the only-Glucantime group.

The anti-microorganism property of clarithromycin is carried out through protein synthesis inhibition by reversibly connecting to 50S ribosomal subunits. It is highly concentrated in phagocytes, effectively transported to the site of infection, and has performance against intracellular microorganisms (Balcioğlu et al., 2012; Sazgarnia et al., 2012). Based on substantial evidence, clarithromycin acts against cutaneous infections, including *Mycobacterium chelonae*, *Corynebacterium minutissimum*, and *Mycobacterium intracellulare*, and has also been used in various skin conditions like rosacea, leprosy, and erythrasma (Sazgarnia et al., 2012; Abokwidir and Feldman, 2016). It was suggested to be effective not only against bacteria but also against protozoa such as *Toxoplasma gondii*, *Cryptosporidium spp*., and *Plasmodium spp*. (Hardman et al., 1996).

Balcioğlu et al. (2012) recommended azithromycin and clarithromycin as possible, effective, and safe therapeutic agents for Leishmania tropica throughout the *in vitro* study with more efficacy in clarithromycin administration. They also suggested that *in vivo* studies should be planned to detect intracellular concentrations of these drugs and determine the effective route and dosage.

Azithromycin has been used in a few research studies for CL with promising results. Possibly, due to its immunomodulatory activity, it can accelerate clinical improvement (Krolewiecki et al., 2002; Sinagra et al., 2007; Amer et al., 2016; Zabolinejad et al., 2020; Abtahi-Naeini et al., 2021). Given the similarity of clarithromycin and azithromycin, a possible therapeutic role in CL for clarithromycin has been proposed (Zabolinejad et al., 2020). Sazgarnia et al. (2012) conducted an *in vitro* study and reported that clarithromycin administration in both liposomal and non-liposomal forms had significant activity against leishmaniasis and suggested that this therapeutic technique should be used in human subjects.

Recently, Zabolinejad et al. (2020) evaluated the effects of oral clarithromycin in combination with systemic Glucantime on 20 patients and reported that this technique significantly reduced lesion size and was considered a safe and effective treatment option. Our results also showed significant effects of liposomal clarithromycin on CL, which is consistent with the previous studies that have reported the efficacy of clarithromycin on CL. The liposomal formulation of clarithromycin has been proposed to be highly effective against sensitive bacteria compared to a free drug formulation (Alhajlan et al., 2013). Additionally, liposomes have advantages like greater skin and intestinal penetration, controlled drug release, localized and limited adverse effects, targeted treatment for skin lesions, and low systemic absorption (Khamesipour et al., 2022; Banerjee et al., 2023).

Considering an increase in the rate of Leishmania resistance, a major treatment policy involves combination therapy, utilizing available drugs through new drug delivery methods such as liposomes (Tiuman et al., 2011; Ponte-Sucre et al., 2017). Other available topical drug formulations, including paromomycin and liposomal amphotericin B, have been investigated and suggested to be effective against CL, but further evaluation is needed (Wolf Nassif et al., 2017; Khamesipour et al., 2022). Macrolides, such as azithromycin and clarithromycin, are among the few suggested drugs with advantages of low risk of toxicity, diverse administration options, and safe usage during pregnancy and childhood (Balcioğlu et al., 2012).

To the best of our knowledge, the present study is the first *in vivo* clinical trial investigating topical liposomal clarithromycin’s effects. Our results align with the previous studies’ results, demonstrating the effectiveness of clarithromycin in reducing the size of lesions in leishmaniasis. In addition, the clarithromycin trial was associated with a faster response in reducing induration. We believe the daily administration of liposomal clarithromycin in combination with systemic Glucantime is a beneficial therapeutic strategy.

Nevertheless, our study had limitations. These include a small sample size, a short follow-up period, and a lack of comparison with other therapeutic protocols, even systemic clarithromycin. A short treatment period with clarithromycin is another limitation to our study, and we could not analyze its efficacy on complete scar formation. As a result, we recommend a large-scale, randomized, controlled trial to evaluate the effectiveness of liposomal and non-liposomal clarithromycin for CL and to compare it with other available treatments with a more prolonged treatment period.

## 5 Conclusion

The present study is the pilot clinical trial that investigates the effectiveness of liposomal clarithromycin. We showed that the administration of liposomal clarithromycin along with systemic Glucantime had a significant beneficial effect on lesion size in CL. These results were in line with the previous studies, but we also suggest that more studies on larger populations should be performed.

## Data Availability

The raw data supporting the conclusion of this article will be made available by the authors, without undue reservation.
